# Supplementary material for: Switch-like Gene Expression Modulates Disease Susceptibility
Source: Res Sq. 2024 Sep 13:rs.3.rs-4974188. Preprint. [Version 1] doi: 10.21203/rs.3.rs-4974188/v1 (PMC11419265; doi:10.21203/rs.3.rs-4974188/v1)
Supplement: Supplement 1 [file NIHPPrs4974188v1-supplement-1.pdf]

Supplementary Information

Principal component analysis on tissue-to-tissue co-expression vectors

We applied a principal component analysis to the 19,132 vectors of tissue-to-tissue co-expression, one vector for each gene. We find that PC1 (**Figure 2A**), explaining 35.3% of the variation, is nearly perfectly correlated with mean tissue-to-tissue co-expression across tissue-tissue pairs (r^2 = 0.998, p-value < 2.2×10^{-16}; **Figure S1**). This result indicates that the 35.3% of the variation in the tissue-to-tissue co-expression of genes is primarily explained by the mean tissue-to-tissue co-expression of genes.

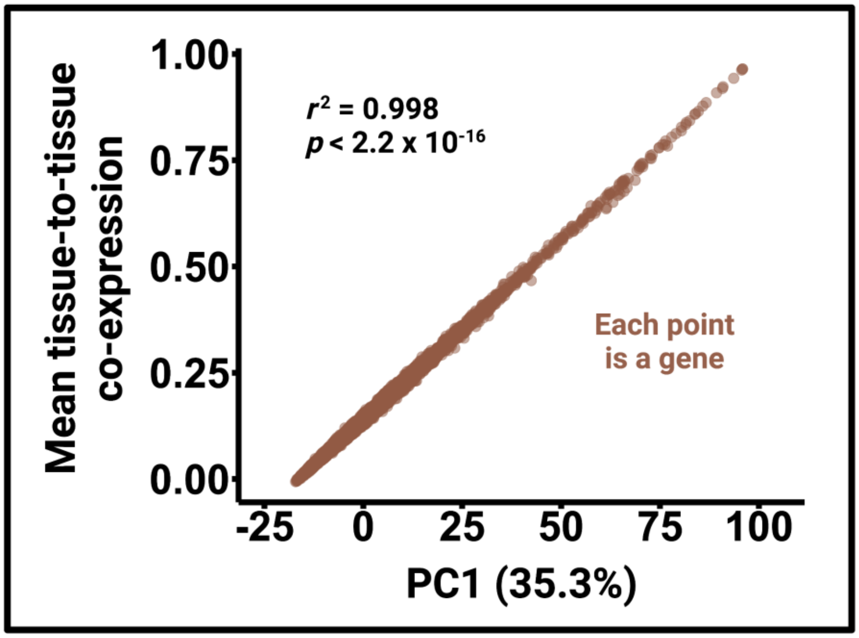

Figure S1. The mean tissue-to-tissue co-expression of genes shows a near-perfect correlation with PC1.

Universally switch-like genes and their biomedical implications

In the main text, we discussed the *USP32P2* and *FAM106A*. Here, we discuss some other interesting examples of universally switch-like genes. The violin plots for the expression level distributions for all cluster-2A and cluster-2B switch-like genes not shown in the main text are present in **Figure S2** and **Figure S3**, respectively.

Firstly, a common ~20kb whole-gene deletion (esv3587154) of the *GSTM1* gene [69,70] is associated with bladder cancer in humans [71]. *GSTM1* is bimodally expressed across individuals in all tissues (**Figure S2D**) that we analyzed, as well as across multiple tumor types [15], with different expression peaks corresponding to differential prognoses among patients. These findings suggest a compelling hypothesis: the common deletion of *GSTM1*, maintained either by drift or balancing selection [72], has no significant effect on the health of non-cancerous individuals; however, it could have significant implications for prognosis once certain types of tumors develop. Therefore, screening

patients with certain tumor types for the *GSTM1* deletion could significantly advance our ability to predict the course of tumor progression in an individualized manner.

Secondly, genes that are bimodally expressed across multiple tissues raise an evolutionary paradox. Typically, genes with a wide expression breadth (i.e., expression across a large number of tissues) affect fitness and are thus constrained at both the sequence and expression level [26,73–75]. However, universally switch-like genes, despite having a high expression breadth, are not conserved at the expression level. This could imply different health consequences for individuals with off versus on state of the genes. For example, the universally switch-like gene RP4-765C7.2 (ENSG00000213058; **Figure S2K**) is upregulated in the peripheral blood mononuclear cells of patients with ankylosing spondylitis [76], eutopic endometrium in endometriosis patients [77], and peripheral blood mononuclear cells of multiple sclerosis patients [78]. Conversely, it is downregulated in the peripheral blood mononuclear cells of Sjögren's syndrome patients [79]. These results suggest that this gene being switched on versus off may predispose individuals to certain diseases while protecting them against others. This balance between susceptibility and protection could explain why both high-expression and low-expression states are maintained in the population at comparable frequencies.

Thirdly, the bimodality of *NPIPA5* (**Figure S2G**)*,* too, can be explained by a single eQTL. The T allele of the SNV rs3198697 is associated with *NPIPA5* being switched on across tissues, while the C allele is associated with the gene being switched off. *NPIPA5* has been reported as one of the top differentially expressed genes among patients with multiple sclerosis in both blood and brain [80]. Moreover, this study [80] showed that this gene is co-expressed in blood and brain. Here, we have shown that this gene is switch-like and that the co-expression of *NPIPA5* is not restricted to blood and brain but extends to all pairs of tissues.

Lastly, a single eQTL can explain the bimodality of a member of the PKD1 gene family in cluster 2A, *PKD1P5* (**Figure S2I**). For *PKD1P5*, the C allele of the SNV rs201525245 is associated with the gene being switched on, while the G allele is associated with the gene being switched off.

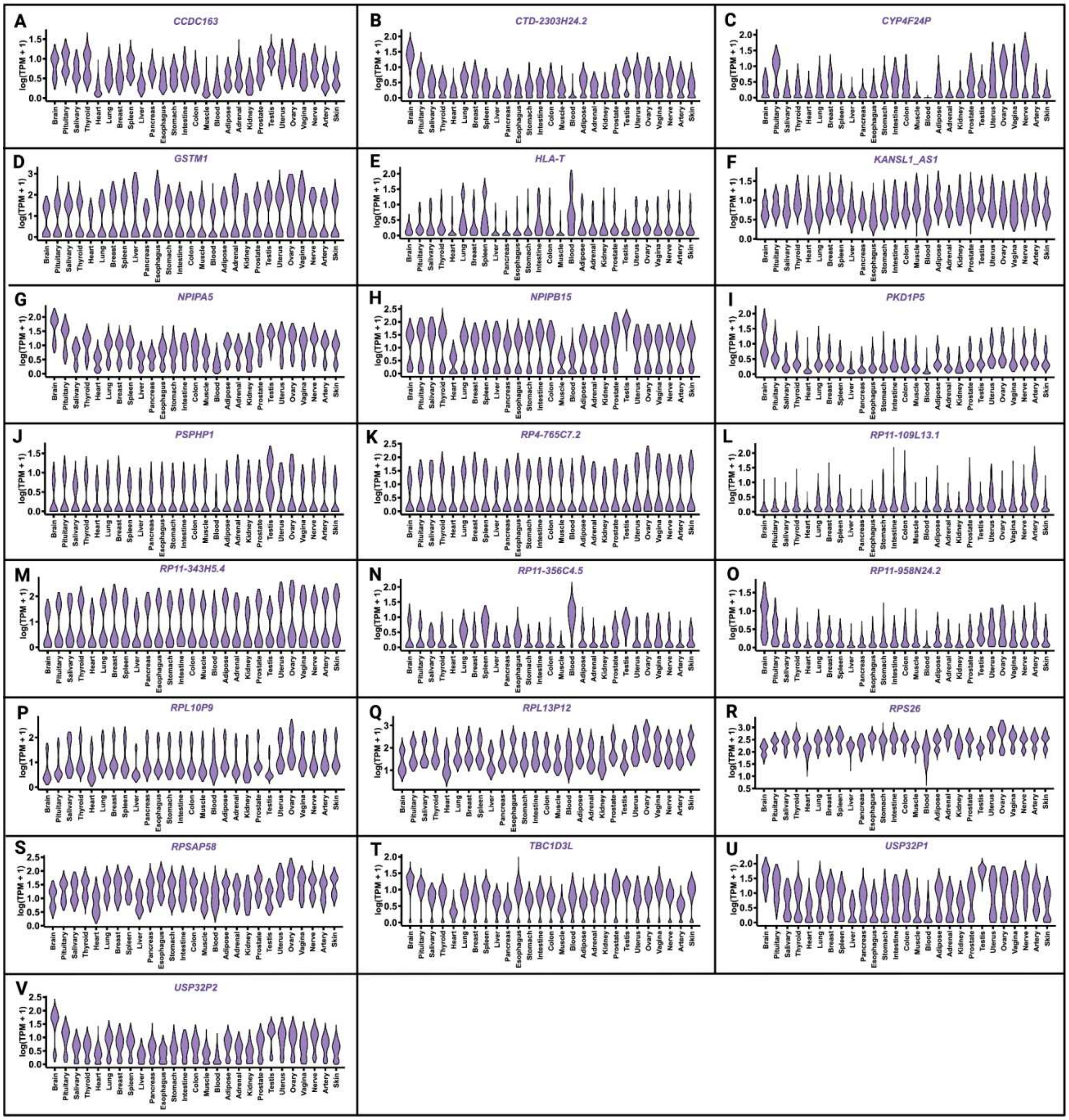

Figure S2. Violin plots for expression level distributions of switch-like genes in cluster 2A.

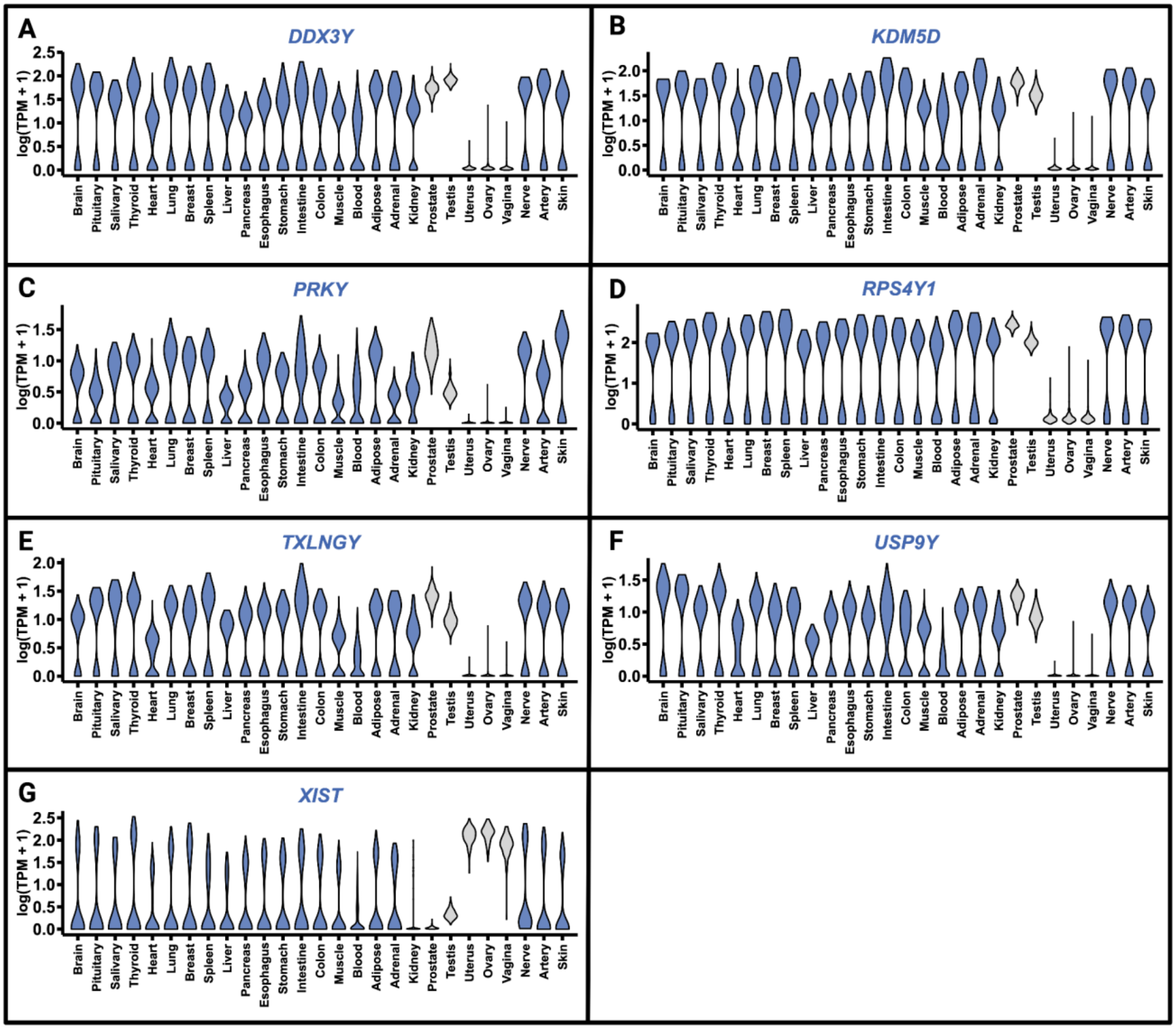

Figure S3. Violin plots for expression level distributions of switch-like genes in cluster 2A.

Conceptual issues regarding bimodal expression distributions driven by genetic polymorphisms

In the main text, we claimed that genetic polymorphisms drive the bimodal expression of universally switch-like genes in cluster 2A. For a polymorphism with two alleles (A and a), there are three possible genotypes (aa, Aa, *and* AA). Since each of the three genotypes can lead to three different expression levels, we expect expression distributions of a cluster-2A gene to have three modes. This leads to the question: Why do we not see trimodal, as opposed to bimodal, expression distributions for genes in cluster 2A? To answer this question, we develop the following frameworks. Let us assume that a genetic polymorphism exists with two alleles, A and a, with frequencies p_A and $(1-p_A)$, respectively. The three genotypes for this polymorphism, aa, Aa, and $AA,$ lead to three different expression states (TPM levels) for the gene with averages

μ_{aa}, μ_{Aa}, and μ_{AA}, respectively. Let us also assume that the Hardy-Weinberg equilibrium holds for this locus. Then, the frequency of aa = $(1-p_A)^2$, the frequency of Aa = $2p_A(1-p_A)$, and the frequency of AA = $p_A{}^2$. We assume that $\mu_{aa} \leq \mu_{Aa} \leq \mu_{AA}$. Next, we define a dominance coefficient $0 \leq \alpha \leq 1$ by,

$$\mu_{Aa} = \mu_{aa} + (\mu_{AA} - \mu_{aa})\alpha.$$

If we define the ratio R by

$$R = \frac{\mu_{AA}}{\mu_{aa}},$$

then, we obtain

$$\mu_{Aa} = \mu_{aa}\,(1 - \alpha + R\alpha)$$

and

$$\mu_{AA} = R\mu_{aa}.$$

We can then divide individuals into three groups depending on their genotypes. Let us assume that the coefficient of variation (CV) of expression is the same for each genotypic group. Then, we can model the TPM value of this gene in a given individual a normal random variable with:

1) mean = μ_{aa} and standard deviation = CV x μ_{aa} if the genotype is aa;
2) mean = μ_{Aa} and standard deviation = CV x μ_{Aa} if the genotype is Aa; and
3) mean = μ_{AA} and standard deviation = CV x μ_{AA} if the genotype is AA.

The value of μ_{aa} is irrelevant for gauging the effect of polymorphisms on the shape of the expression level distributions. Therefore, we set $\mu_{aa} = 1$.

Under these mathematical assumptions, we performed simulations using 36 distinct models. These models vary by four parameters: $p_A \in \{0.05, 0.1, 0.5\}$, CV $\in \{0.1, 0.3\}$, $R \in \{10, 1000\}$, and $\alpha \in \{0.2, 0.5, 0.8\}$. For each model, defined by a unique combination of the values of these four parameters, we performed a two-step sampling procedure. First, we obtained a random sample of 500 genotypes, based on p_A and the Hardy-Weinberg equilibrium. Next, for each of the 500 genotypes sampled, we sample a TPM value from the normal distribution corresponding to that genotype. Thus, for each of the 36 models, we simulated 500 TPM values. We present these values as histograms with and without log transformation. The results for $p_A = 0.05$, $p_A = 0.1$, and $p_A = 0.5$ are shown in **Figure S4**, **Figure S5**, and **Figure S6**, respectively**.** These simulations help us answer our question we first asked: Why do we not see a trimodal distribution if a genetic polymorphism drives expression-level variability in a gene?

Firstly, even when the minor allele (A) frequency is not low (e.g., 10%), the frequency of the genotype AA is still quite low (e.g., 1%). Therefore, the third peak is not always conspicuously visible. We see this in all models with $p_A = 0.05$ and $p_A = 0.1$ (**Figures S4** and **S5**), regardless of CV, R, and α values. At higher allele frequencies (e.g., 50%), the effect of the remaining parameters becomes more apparent. **Figure S6** shows that a

higher dominance coefficient α makes the expression level distribution more bimodal. By contrast, a lower dominance coefficient α makes the expression level distribution more trimodal. The lack of observed trimodality in the GTEx data may suggest that expression levels of switch-like genes tend to be more dominant than additive with regard to causal genetic polymorphisms. Secondly, greater variation (CV) in the data can also obscure the third peak. For example, by comparing **Figure S6B** to **Figure S6H**, we find that increasing the CV can change the distribution from being trimodal to bimodal when the other parameters are held constant. However, R does not seem to have much effect on whether the expression level distribution is bimodal or trimodal.

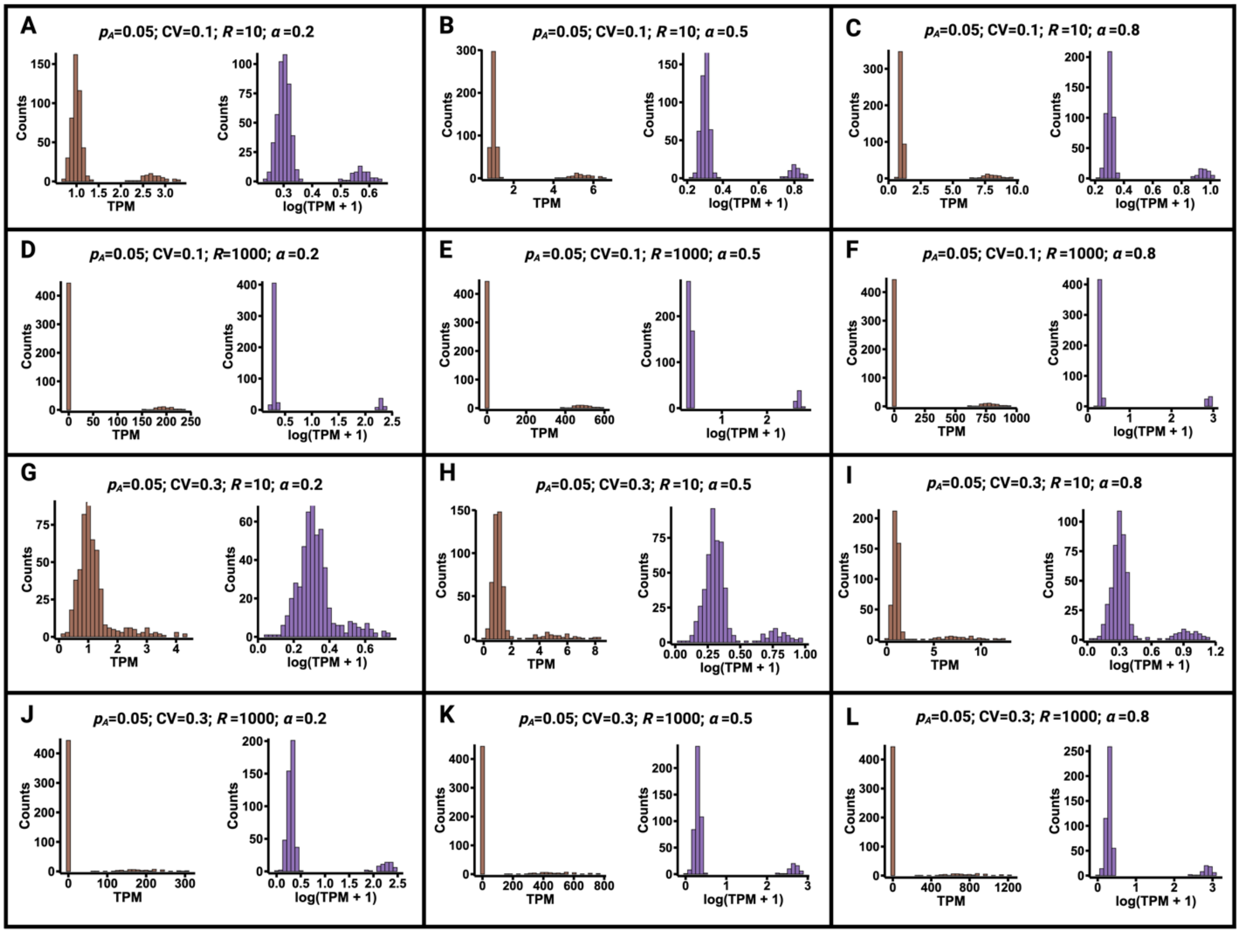

Figure S4. TPM simulations for a hypothetical gene whose expression is driven by a genetic polymorphism with an allele frequency of 5%.

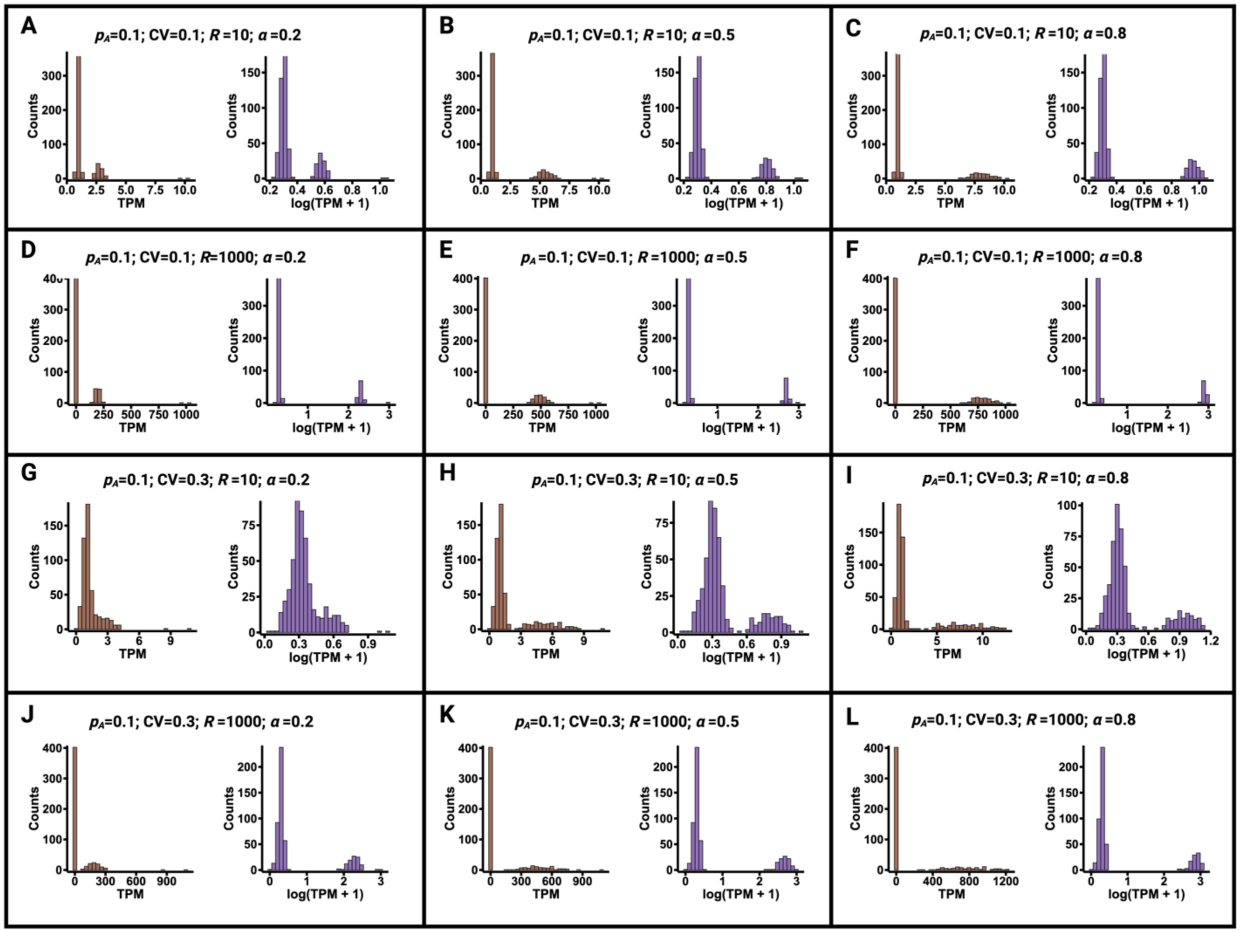

Figure S5. TPM simulations for a hypothetical gene whose expression is driven by a genetic polymorphism with an allele frequency of 10%.

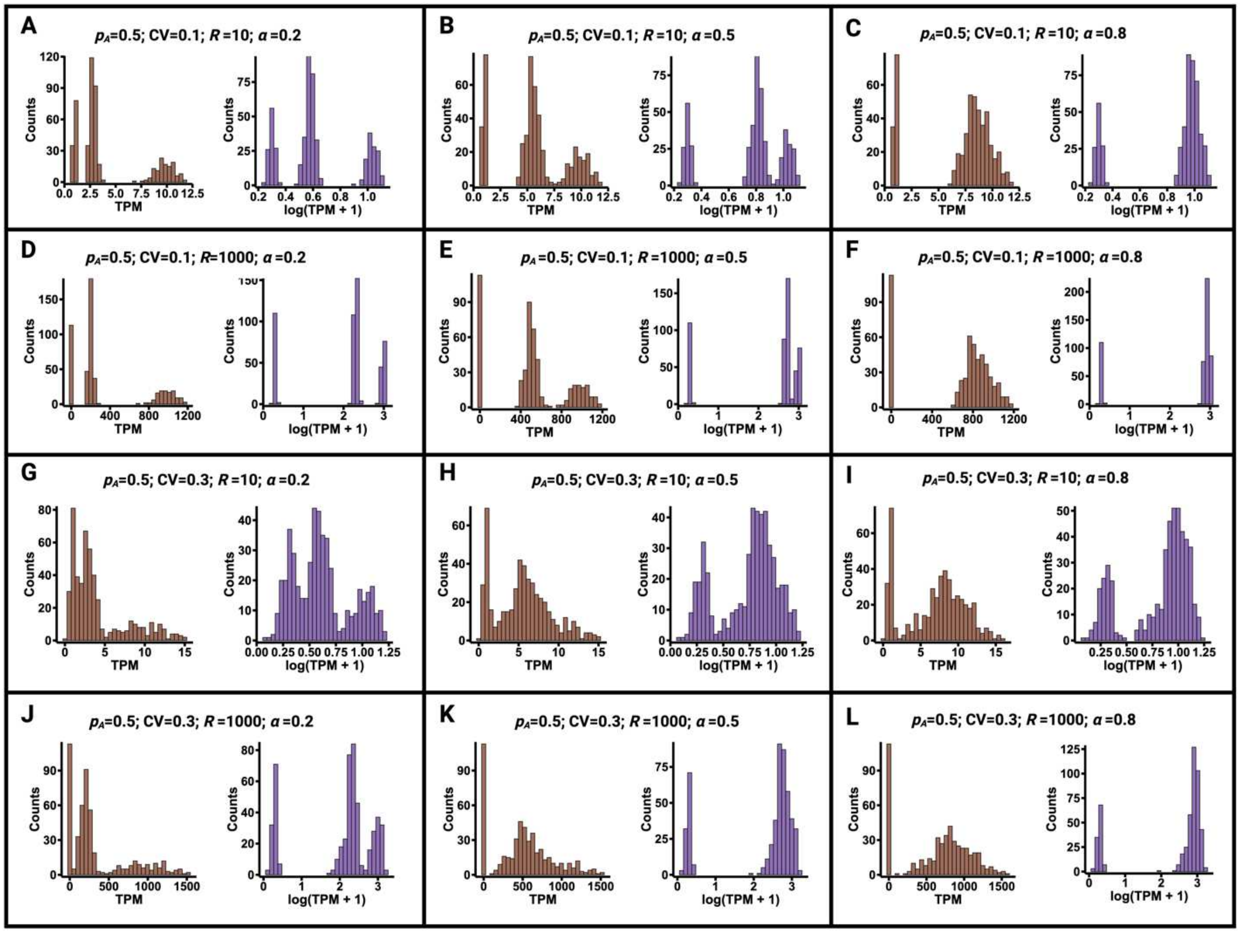

Figure S6. TPM simulations for a hypothetical gene whose expression is driven by a genetic polymorphism with an allele frequency of 50%.

Tissue-specific switch-like genes

We divided switch-like genes into three clusters in the space spanned by the first two principal components (**Figure 2A**). While we said that genes in cluster 1 **(Figure 2A-B)** are tissue-specific switch-like genes, manual inspection reveals this is not true for all genes in cluster 1. In particular, the transcript ENSG00000273906 coming from chr Y was labeled cluster 1 by hierarchical clustering even though it is universally switch-like in tissues common to both sexes. Indeed, we removed all chr-Y genes from our analyses of genuine cluster-1 genes. Other cluster-1 genes bimodally expressed in a large number of tissues lie on the autosomes. For example, *CLPS*, *PRSS1*, *CELA3A*, and *CELA3B*, despite having low overall tissue-to-tissue co-expression, are bimodally expressed across tissues. Indeed, we have shown previously that *CELA3A* and *CELA3B* have a shared regulatory architecture in the pancreas [81].

Controlling for confounders

We removed cluster-1 genes affected by confounders in each tissue using an approach outlined in **Methods**. Here, we present the number of genuine cluster-1 genes versus

those affected by confounders in **Figure S7**. In particular, we show that the cluster-1 genes in the colon and the intestine are particularly prone to being affected by confounding factors. We also present in **Figure S8** examples of genes whose bimodal expression in specific tissues is correlated with variation in the sample ischemic time distribution.

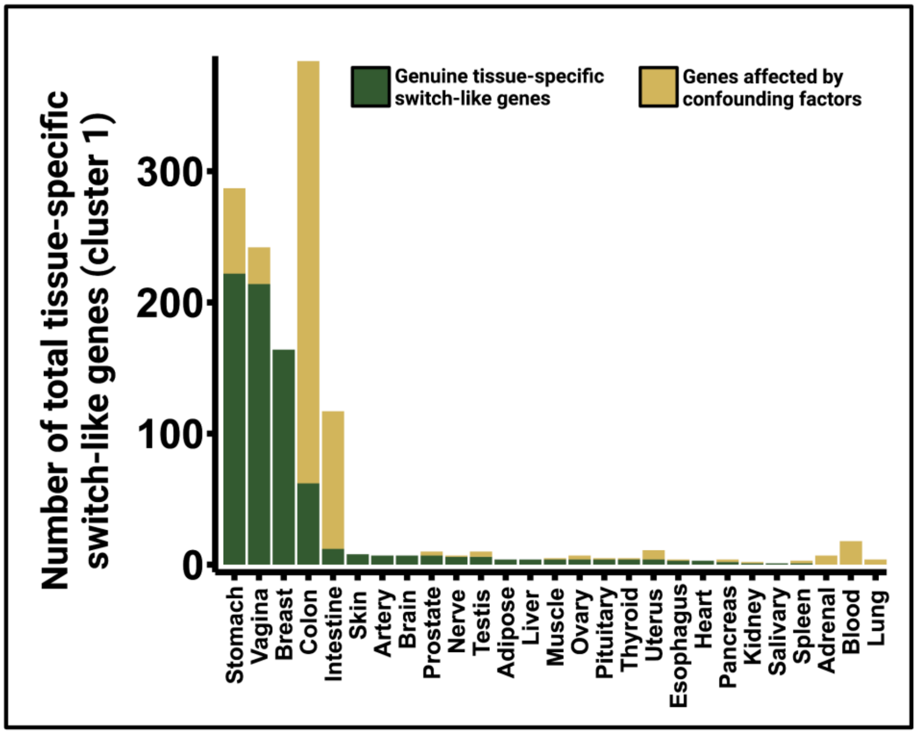

Figure S7. Switch-like genes in cluster 1 that are genuine versus those affected by confounders.

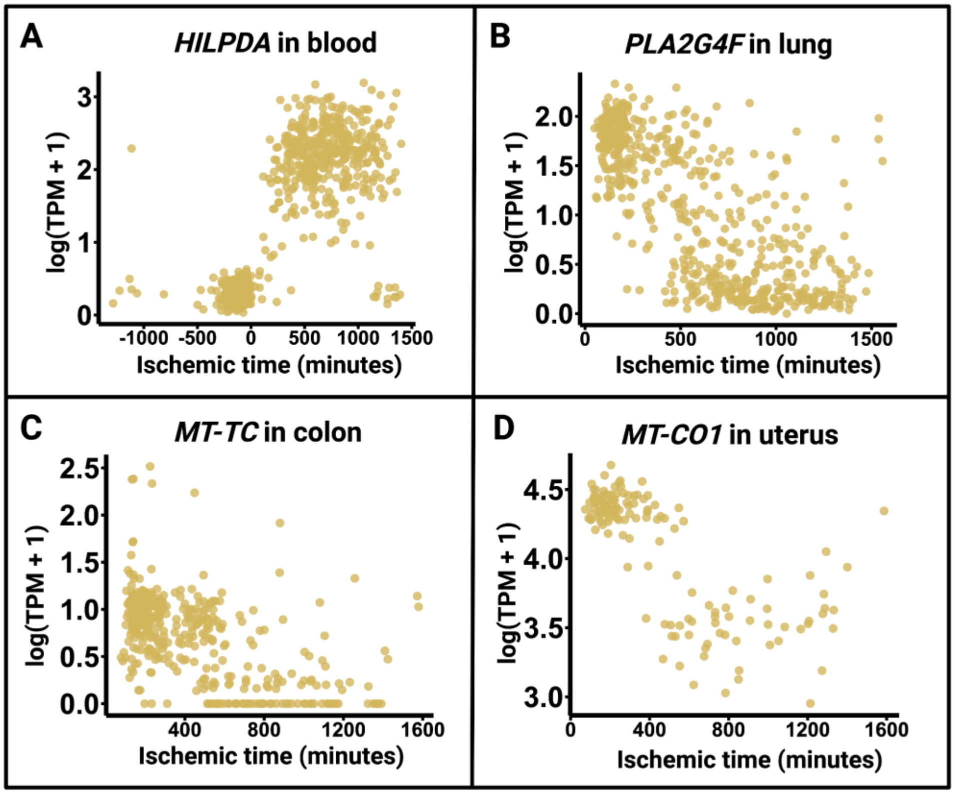

Figure S8. Examples of cluster-1 genes affected by confounders. Their bimodal distribution is caused by ischemic time (a confounding factor).

The copy number variation at the *PGA3* locus does not affect the gene's expression levels

PGA3 exhibits a high copy number variation among humans [82], but the copy number seems to have no impact on *PGA3* expression, at least in cancer samples [83]. The bimodal expression of PGA3 in the stomach is likely not due to its copy number variation. This is because PGA3's expression in the stomach is highly correlated with other tissue-specific genes in the stomach. The only way in which a copy number-driven bimodality of PGA3 could be correlated with other switch-like genes is if the product of PGA3 was regulating the correlated genes. Without this evidence, we surmise that the copy number variation at the PGA3 locus does not affect the gene's expression levels, at least in the stomach.

Table S1. A list of tissues used in this study along with the number of individuals for each tissue.
Table S2. A list of 1,013 switch-like genes.
Table S3. Tissue-to-tissue co-expression (Pearson's correlation) for all genes across 310 tissue-tissue pairs.
Table S4. Results from principal component analysis on tissue-to-tissue co-expression data for all genes.
Table S5. Results from principal component analysis on tissue-to-tissue co-expression data for only switch-like genes.
Table S6. Correlation between gene expression levels and confounding factors for switch-like

genes.
Table S7. Gene-to-gene co-expression of genuine tissue-specific switch-like genes in the stomach, vagina, breast, and colon.
Table S8. Analysis of sex bias among genuine tissue-specific switch-like genes.
